# Supplementary material for: The TUTase URT1 connects decapping activators and prevents the accumulation of excessively deadenylated mRNAs to avoid siRNA biogenesis
Source: Nat Commun. 2021 Feb 26;12:1298. doi: 10.1038/s41467-021-21382-2 (PMC7910438; doi:10.1038/s41467-021-21382-2)
Supplement: Supplementary file 3 — Descriptions of Additional Supplementary Files [file 41467_2021_21382_MOESM3_ESM.pdf]

Descriptions of Additional Supplementary Files

Supplementary Data 1

Description: Sequences used for phylogeny analysis, Related to Fig. 1.

Supplementary Data 2

Description: Differential analysis of protein accumulation in URT1 IPs as compared to control or to m1 URT1 IPs, Related to Fig. 2 and Supplementary Fig. 3. a,b Differential analysis of protein accumulation in URT1 IPs as compared to control IPs. Immunoprecipitation experiments were performed with (a) or without (b) formaldehyde crosslink. c Differential analysis of protein accumulation in m1URT1 IPs as compared to URT1 IPs. d Details of biological material used for IPs.

Supplementary Data 3

Description: Source data related to 3'RACE-seq experiments, Related to Fig. 3-5 and to Supplementary Fig. 3-5. Number of reads analyzed at each step of the data processing for N. benthamiana (a,b) and A. thaliana (c,d).

Supplementary Data 4

Description: Source data related to nanopore DRS experiments, Related to Fig. 5a. Summary of the number of reads obtained for nanopore DRS.

Supplementary Data 5

Description: Source data related to TAIL-seq experiment, Related to Fig. 5d and 6f. a Summary of the number of reads obtained at each step of the data processing step. b Uridylation frequency for the 3440 detected mRNAs.

Supplementary Data 6

Description: Source data related to small RNA-seq experiment, Related to Fig. 6. a Summary of the number of reads obtained for small RNA-seq. b Total number of 21-25 nt reads that map on mRNAs in WT, urt1-1, xrn4-3 and urt1-1 xrn4-3. c List of mRNA loci that show differential siRNA accumulation in urt1-1, xrn4-3, urt1-1 xrn4-3 when compared to WT.

Supplementary Data 7

Description: List of primers and plasmids used in this study
