# Supplementary material for: The TUTase URT1 connects decapping activators and prevents the accumulation of excessively deadenylated mRNAs to avoid siRNA biogenesis
Source: Nat Commun. 2021 Feb 26;12:1298. doi: 10.1038/s41467-021-21382-2 (PMC7910438; doi:10.1038/s41467-021-21382-2)
Supplement: Supplementary file 11 — Reporting Summary [file 41467_2021_21382_MOESM11_ESM.pdf]

nature research

Corresponding author(s): Dominique Gagliardi & Hélène Zuber

Last updated by author(s): Jan 7, 2021

Reporting Summary

Nature Research wishes to improve the reproducibility of the work that we publish. This form provides structure for consistency and transparency in reporting. For further information on Nature Research policies, see our Editorial Policies and the Editorial Policy Checklist.

Statistics

For all statistical analyses, confirm that the following items are present in the figure legend, table legend, main text, or Methods section.

n/a	Confirmed	
☐	☒	The exact sample size (*n*) for each experimental group/condition, given as a discrete number and unit of measurement
☐	☒	A statement on whether measurements were taken from distinct samples or whether the same sample was measured repeatedly
☐	☒	The statistical test(s) used AND whether they are one- or two-sided<br>*Only common tests should be described solely by name; describe more complex techniques in the Methods section.*
☒	☐	A description of all covariates tested
☐	☒	A description of any assumptions or corrections, such as tests of normality and adjustment for multiple comparisons
☐	☒	A full description of the statistical parameters including central tendency (e.g. means) or other basic estimates (e.g. regression coefficient) AND variation (e.g. standard deviation) or associated estimates of uncertainty (e.g. confidence intervals)
☐	☒	For null hypothesis testing, the test statistic (e.g. *F*, *t*, *r*) with confidence intervals, effect sizes, degrees of freedom and *P* value noted<br>*Give P values as exact values whenever suitable.*
☒	☐	For Bayesian analysis, information on the choice of priors and Markov chain Monte Carlo settings
☒	☐	For hierarchical and complex designs, identification of the appropriate level for tests and full reporting of outcomes
☒	☐	Estimates of effect sizes (e.g. Cohen's *d*, Pearson's *r*), indicating how they were calculated

Our web collection on statistics for biologists contains articles on many of the points above.

Software and code

Policy information about availability of computer code

Data collection	Illumina sequencing: HiSeq Control Software 2.2.38, MiSeq Control Software 2.6.2.1<br>Image vizualisation : Amersham Typhoon Scanner v1.0.0.7, ImageJ 1.52a, Attan DIGE imager v1.0 (Amersham Biosciences), Fusion FX camera system (2013, Vilber Lourmat)<br>LCMSMS : XCalibur v4.3 (QExactive+), Analyst v 1.6 (TripleTOF5600)<br>Nanopore sequencing : MinKNOW Core v3.6.5
Data analysis	Proteomic :<br>- Raw data processing : Proteome Discoverer software (v2.3, Thermo Scientific);<br>- Peptide and protein identification (database search): Mascot (version 2.5, Matrix Science)<br>- Peptide and protein validation, Spectral Count quantification and samples alignment: Proline 1.4 software.<br>- Differential analysis based on spectral counts: IPinquiry R package v4.5 (https://github.com/hzuber67/IPinquiry4),<br>Illumina sequencing:<br>- HiSeq Control Software 2.2.38, RTA 1.18.61.0, CASAVA-1.8.2 (Illumina) ;<br>- MiSeq Control Software 2.6.2.1 MiSeq Reporter 2.6.2.3<br>- Mapping and analysis: Bowtie v1.0.0, Bowtie 2 v2.3.5, Hisat2 v2.1.0, BEDTools suite v2.17.0,<br>- TAILseeker software v3.1<br>Statistic analyses :<br>- R 3.6.1, Rstudio 1.2<br>- R packages : edgeR 3.26.5, stats 3.6.1, multcompView 0.1-8.<br>Phylogenetic analysis : BLASTP (NCBI, 2019), TBLASTN (NCBI, 2019), Expasy translate tool (2019), MUSCLE v3.8.31, PhyML v. 3.1, Gblocks 0.91b, FELLS v1.1,

Jalview 2.10.5, iTOL 5, SUPERFAMILY 2.0, WebLogo3, fLPS, FELLS v1.1
Gene ontology analysis : DAVID 6.8
Nanopore sequencing : Guppy 4.0.11 (Oxford Nanopore Technologies), MiniMap 2.17, Nanopolish 0.13.2
Quantification of CAF1b activity : ImageQuanTL software v8.1
Quantification of GFP fluorescence: ImageJ v1.52
3'RACE-seq pipelines : python (v2.7), biopython (v1.63), regex (v2.4). Python and bash source code are available as Mendeley data at http://dx.doi.org/10.17632/v8d9bd692c.1

For manuscripts utilizing custom algorithms or software that are central to the research but not yet described in published literature, software must be made available to editors and reviewers. We strongly encourage code deposition in a community repository (e.g. GitHub). See the Nature Research guidelines for submitting code & software for further information.

Data

Policy information about availability of data

All manuscripts must include a data availability statement. This statement should provide the following information, where applicable:

- Accession codes, unique identifiers, or web links for publicly available datasets
- A list of figures that have associated raw data
- A description of any restrictions on data availability

NGS datasets generated during this study have been deposited in NCBI's Gene Expression Omnibus70 and are accessible through GEO Series accession number GSE148449. GEO Series accession numbers for individual datasets are GSE148406 for 3' RACE-seq in Arabidopsis, GSE148409 for 3'RACE-seq in N. benthamiana, GSE148417 for TAIL-seq and GSE148427 for small-RNA seq.
Mass spectrometry proteomics raw data have been deposited to the ProteomeXchange Consortium via the PRIDE partner repository71 with dataset identifiers PXD018672 (10.6019/PXD018672) and PXD022676 (10.6019/PXD022676) for Arabidopsis and N. benthamiana, respectively.
Raw data for Nanopore DRS have been deposited at ENA with the accession number PRJEB40438.
Source data for all figures in the paper, including raw data underlying graphs and uncropped versions of gels or blots presented in the figures, are available as Mendeley data: http://dx.doi.org/10.17632/ybcvvmtcn9.2.
The raw intensity files (.cif files) used to test the TAILseeker3 software (results shown in Supplementary Fig. 2) have not been deposited in a public repository because of their large size but are available from the corresponding author on request.
Web links for associated raw data are indicated in each figure legends.

Field-specific reporting

Please select the one below that is the best fit for your research. If you are not sure, read the appropriate sections before making your selection.

☒ Life sciences ☐ Behavioural & social sciences ☐ Ecological, evolutionary & environmental sciences

For a reference copy of the document with all sections, see nature.com/documents/nr-reporting-summary-flat.pdf

Life sciences study design

All studies must disclose on these points even when the disclosure is negative.

Sample size	No sample-size calculation was performed. The sample size was determined based on the cost, the complexity and sufficient statistical power of the experimental approach and takes into account the technical and biological variability. The sample size was similar to the one typically used for similar approaches.
Data exclusions	No data was excluded
Replication	All experimental findings were reproduced in independent replicates. The number of independent replicates is indicated in the figure or in the corresponding figure legend. In addition, most experimental findings were validated using alternative experimental approaches.<br>Of note, in our manuscript, biological replicates refer to plants of the same genotype grown at different times. In addition, some biological replicates originated from a different seed stock or an independent mutagenesis event.
Randomization	Due to the nature of experimental setup, randomization is not reasonably applicable
Blinding	Due to the nature of experimental setup, blinding is not reasonably applicable

Reporting for specific materials, systems and methods

We require information from authors about some types of materials, experimental systems and methods used in many studies. Here, indicate whether each material, system or method listed is relevant to your study. If you are not sure if a list item applies to your research, read the appropriate section before selecting a response.

Materials & experimental systems

n/a	Involved in the study
☐	☒ Antibodies
☒	☐ Eukaryotic cell lines
☒	☐ Palaeontology and archaeology
☒	☐ Animals and other organisms
☒	☐ Human research participants
☒	☐ Clinical data
☒	☐ Dual use research of concern

Methods

n/a	Involved in the study
☒	☐ ChIP-seq
☒	☐ Flow cytometry
☒	☐ MRI-based neuroimaging

Antibodies

Antibodies used	Mouse monoclonal anti-c-Myc (Roche, 11667203001)<br>Rabbit polyclonal anti-MBP (Invitrogen, PA1-989)<br>Goat anti-Mouse IgG (H+L) (Invitrogen G-21040)<br>Goat anti-Mouse IgG (H+L) (Invitrogen, G-21040)<br>Goat anti-Rabbit IgG (H+L) (Invitrogen, G-21234)<br>Rabbit polyclonal anti-URT1 (Zuber et al. 2016)<br>Rabbit polyclonal anti-DCP5 (provided by Rémy Merret and Cécile Bousquet-Antonelli , Perpignan, France)
Validation	In our study, antibodies were used for western analyses. All western were performed with appropriate controls. For commercial antibodies, please also see product information on the corresponding website.
